# First Report of *Enterobacter ludwigii* and Other Potentially Pathogenic Enteric Bacteria in Onions, Soil, and Irrigation Water from the Vhembe Region, South Africa

**DOI:** 10.3390/foods15173046

**Published:** 2026-08-28

**Authors:** Afsatou Ndama Traoré, Elelwani Lukheli, Damien Georges Jacobs, Ceryl Mphedziseni Mampheu, Tiisetso Colleen Maphaisa, Natasha Potgieter

**Affiliations:** Biochemistry and Microbiology Department, University of Venda, Thohoyandou 0950, South Africa; 17008303@mvula.univen.ac.za (E.L.); 17017009@mvula.univen.ac.za (D.G.J.); 19012109@mvula.univen.ac.za (C.M.M.); tiisetso.maphaisa@univen.ac.za (T.C.M.); natasha.potgieter@univen.ac.za (N.P.)

**Keywords:** antimicrobial resistance, onions, irrigation water, agricultural soil, *Enterobacter ludwigii*, *Escherichia coli*, 16S rRNA sequencing

## Abstract

The presence of antimicrobial-resistant bacteria in fresh produce constitutes a significant public health concern, particularly in rural areas where untreated water is commonly used for irrigation. Certain *Enterobacter* species have been reported as causal agents of onion bulb rot, with *Enterobacter cloacae* experimentally demonstrated to cause bulb rot in onions. However, there is poor documentation of its effects in the Vhembe District, South Africa. This study investigated the detection and identification of enteric bacteria in onions, soil, and irrigation water, as well as the characterisation of the identified isolates. Thirty-six samples were analysed, comprising 13 onion samples, 4 irrigation water samples, and 9 soil specimens collected from three farms using selective and differential agar. Identification and enumeration in water and onion samples were performed with the Colilert Quanti-Tray. Physicochemical analysis of irrigation water indicated that Farm 3 had the highest electrical conductivity (EC) and total dissolved solids (TDS), as well as the lowest pH, while Farm 2 exhibited the greatest diversity of pathogenic *Escherichia coli* pathotypes, including enteroinvasive *E. coli* (EIEC), which was absent in Farm 3. Culture-based methods yielded 72 presumptive bacterial isolates, which were characterized using the VITEK 2 system. Identified species included *Pseudomonas aeruginosa*, *Klebsiella pneumoniae*, *Klebsiella oxytoca*, *Citrobacter amalonaticus*, *Raoultella ornithinolytica*, and members of the *Enterobacter cloacae* complex. Phylogenetic analysis revealed that most Enterobacter isolates closely matched reference strains of *Enterobacter ludwigii*. Antimicrobial susceptibility testing against 19 antibiotics demonstrated varied resistance patterns among the isolates. The *Enterobacter cloacae* complex exhibited the highest resistance (10/19 antibiotics), followed by *Klebsiella pneumoniae* (9/19) and *Citrobacter amalonaticus* (7/19). All isolates were resistant to colistin but remained susceptible to ciprofloxacin, with most also sensitive to gentamicin and amikacin. Future research should include additional farms and investigate antibiotic resistance at the genetic level to elucidate its origins.

## 1. Introduction

*Enterobacter* species have been identified as plant associates, functioning as endophytes, rhizosphere inhabitants, plant growth-promoting bacteria, and, in some cases, plant pathogens [1]. Commonly associated species include *Enterobacter cloacae*, *Enterobacter asburiae*, *Enterobacter hormaechei*, *Enterobacter kobei*, *Enterobacter mori*, and *Enterobacter ludwigii*. Certain strains promote plant growth, nutrient uptake, and biocontrol, whereas others contribute to plant diseases such as soft rot, bulb decay, and internal tissue degradation in various crops [1]. These species have been isolated from a wide range of plant hosts, including maize, alfalfa, pepper, grapevine, onion, and other economically significant crops, highlighting their diverse ecological functions in plant-associated environments and the wide array of potential hosts [2]. Differentiating species within this group could help detect pathogens contributing to the loss of essential crops, identify candidates for future research, and contribute to the growing body of knowledge on enteric bacteria in one of South Africa’s agricultural hubs, the Vhembe Region.

Onion production is increasing worldwide, making food quality standards for vegetables more important and difficult to maintain. Onions are widely consumed in many developing countries, such as South Africa and India, and play a significant role in their economies. In South Africa, onions contribute 2.3 billion to the total gross value of vegetables [3,4,5]; however, onion bulb rot causes major production losses due to fungal and bacterial diseases. Bacteria such as *P. aeruginosa* and members of the Enterobacteriaceae, which are primarily gut bacteria, have been linked to these onion rot infections. Such enteric bacteria include *Pantoea*, *Klebsiella*, and the *Enterobacter cloacae* complex [4,6]. Monitoring these pathogens in staple foods such as onions is especially important in rural farming communities like those in Vhembe, where manure is used in agriculture.

Several factors contribute to bacterial infections in onions, which can be grouped as pre-harvest or post-harvest factors. Pre-harvest factors include improper irrigation, soil type and moisture, and high nitrogen fertilizer use. Post-harvest factors include poor handling and storage, as well as the presence of pathogens during harvest, storage, and handling [4,7]. Enterobacteriaceae are mostly opportunistic pathogens that can cause gastroenteritis as well as diseases like bacteraemia and urinary tract infections.

Antibiotic resistance makes these bacterial diseases spread more easily and become more severe [8]. In the Vhembe District Municipality (VDM), researchers have detected various Enterobacteriaceae in rivers, farm animals, surfaces, and clinical samples [7,9,10]. People in Vhembe rely on onions in their cooking, using them in dishes such as relishes, salads, salsas, and stews. Most onion farming in Vhembe is self-sufficient, with only a few commercial farms. Previous studies show that rivers support many human activities, especially agriculture, which can lead to crop contamination with enteric bacteria [9,11].

Little information exists on the presence of Enterobacteriaceae, particularly in onions, soil, and irrigation water, in the Vhembe District. This study is the first to report on the presence of Enterobacteriaceae on onions, in soil, and in irrigation water from farms in Vhembe, with a focus on Enterobacter.

## 2. Methodology

### 2.1. Study Area

**Figure 1:**
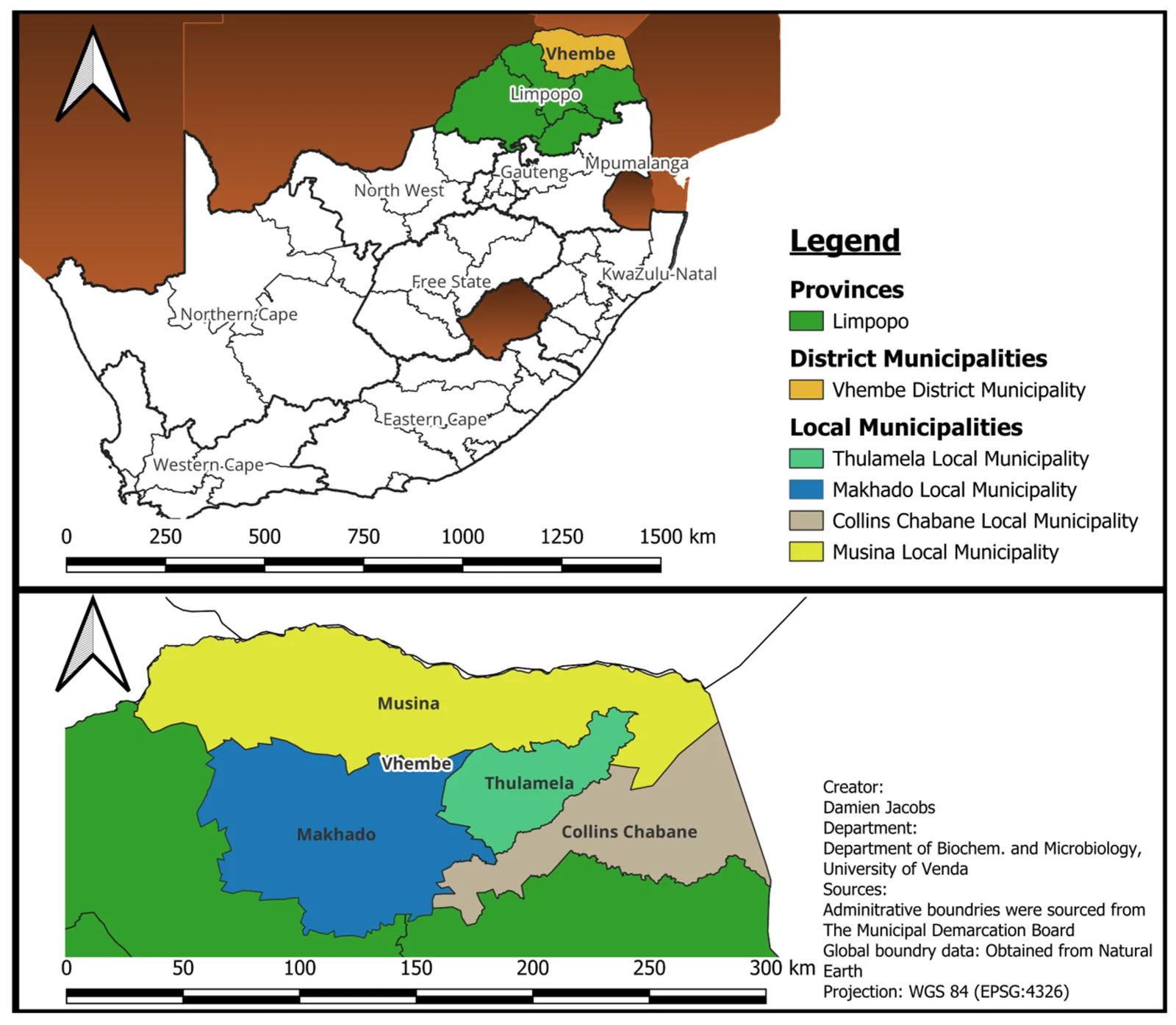
The Vhembe District Municipality and its four local municipalities.

The VDM includes four local municipalities: Collins Chabane, Makhado, Musina, and Thulamela (see Figure 1). The district covers 21,407 km^2^ and is home to over 1.1 million people in 274,480 households.

The study included two subsistence farms that use river water for irrigation and one commercial farm that uses borehole water to irrigate onions. The commercial farm also produces melons and peppers. Farm locations are not disclosed at the request of the farmers.

### 2.2. Sample Collection

We collected 36 samples using sterile gloves (LASEC^®^, Johannesburg; GA, South Africa). Onions came from backyard farms (n = 8) and a commercial farm (n = 5). Soil samples, each weighing 5–10 g, were collected from onion beds at backyard farms (n = 4) and the commercial farm (n = 5) and placed in sterile plastic containers. Water samples (500 mL) were collected in sterile bottles from rivers near informal farms used for irrigation (n = 10), as well as from sprinklers at the commercial farm (n = 3) and a borehole (n = 1). We measured physical parameters, including temperature, total dissolved solids, and electrical conductivity, on-site with a Crison MM40 multi-parameter reader (Crison, Barcelona, Spain).

All onion, water, and soil samples were placed in cooler boxes, sterilized with 70% ethanol and a 3% hypochlorite solution, packed with ice, and transported immediately to the University of Venda’s General Microbiology Research Laboratory for further analysis.

### 2.3. Isolation of Enterobacteriaceae Species and Other Enteric Bacteria

#### 2.3.1. Water Samples

A 100 mL sample was collected from each water source and filtered through a sterile membrane with a 0.45 µm pore size (Sartorius Stedim Biotech GmbH, Gottingen, Germany). The filter was placed on EMB agar plates (Davies Diagnostics, Johannesburg, GA, South Africa). The process was repeated, and a second filter was placed on MacConkey agar plates (Davies Diagnostics, Johannesburg, GA, South Africa) using sterile forceps. Both plates were incubated at 37 °C for 24 h (Inco Therm digital, Labotec; Midrand, GA, South Africa). After incubation, purified colonies were subcultured onto the same type of agar.

The Colilert Quanti-Tray system (IDEXX, Westbrook, ME, USA) was used to detect total coliforms and *E. coli* in the water samples by determining the most probable number (MPN) per 100 mL. In short, a 100 mL water sample was placed in a sterile vessel (IDEXX), and Colilert-18 reagent was added and mixed until dissolved. The mixture was poured into a Quanti-Tray, sealed with a Quanti-Tray sealer (Model 2020, IDEXX), and incubated at 35 °C for 18 h. We included positive controls, including *E. coli* Quanti-Cult reference strains, and processed blank water samples alongside the test samples [12]. After incubation, wells with no colour change, appearing less yellow than the comparator, were considered negative for total coliforms. Wells that turned yellow or were more intensely yellow than the comparator were considered positive for total coliforms. Wells that fluoresced under UV light (Spectroline CM UV; Sigma-Aldrich, St. Louis, MI, USA) at 365 nm were identified as positive for *E. coli*, following the manufacturer’s guidelines [12].

#### 2.3.2. Onion Samples

The scales from onion bulbs were surface sterilised by soaking in 10% ethanol (Merck; Darmstadt, Germany) for 30 s, followed by three rinses in sterile water. The onions were then sliced into small pieces measuring 1 to 5 mm. These tissue pieces were macerated in 200 mL PBS solution (Merck; Darmstadt, Germany) for 5 min. One hundred millilitres of the resulting solution was vortexed, and the streak plate and pour plate methods were used to culture on EMB (Eosin Methylene Blue) and MacConkey agar plates (Davies Diagnostics, Johannesburg, GA, South Africa). These plates were then incubated at 37 °C for 24 h. To purify the colonies, subcultures were performed after the initial incubation on EMB medium (Davies Diagnostics), followed by an additional 24 h incubation at 37 °C. To determine if *E. coli* and total coliforms could be enumerated using biochemical methods from the onions, the remaining portion of the onion sample was vortexed. The onion was removed using sterile forceps, and the suspension was mixed with Colilert Quanti-Tray-18 reagent (IDEXX, Westbrook, ME, USA). The mixture was then poured into a Quanti-Tray, sealed and incubated at 37 °C for 24 h as recommended by the manufacturer [12]. Bacterial counts were not normalized.

#### 2.3.3. Soil Samples

Soil samples were sieved and mixed with 100 mL PBS for 5 min, then vortexed using a Vortex-Genie 2 mixer (LASEC^®^, Johannesburg, GA, South Africa). The liquid was poured onto EMB and MacConkey agar plates (Davies Diagnostics) using the pour plate method, then streaked. Plates were incubated at 37 °C for 24 h using an Inco Therm digital incubator (Labotec, Johannesburg, GA, South Africa). After incubation, isolated colonies were sub-cultured onto fresh plates of the same medium using the quadrant streak method.

### 2.4. Characterization of the Bacterial Species and Their Identification

Following Gram staining, bacterial colonies were examined for their macroscopic and microscopic morphological characteristics according to the modified method of Williamson [13]. Individual colonies displaying distinct morphological features were subsequently isolated and subcultured on nutrient agar (Davies Diagnostics, Johannesburg, GA, South Africa) to obtain pure cultures.

Bacterial isolates were subsequently identified according to their biochemical characteristics using the VITEK^®^ 2 Compact automated identification system (bioMérieux, Marcy-l’Étoile, Rhône-Alpes, France). Presumptive positive pure colonies were suspended in sterile saline and adjusted to the manufacturer-recommended turbidity. These suspensions were inoculated into the appropriate VITEK^®^ 2 identification cards and analyzed following the manufacturer’s instructions. Species identification was performed automatically by the VITEK^®^ 2 software version 9 based on the biochemical reaction profiles generated during incubation.

### 2.5. Antimicrobial Susceptibility Testing

Antimicrobial susceptibility testing of the identified bacterial isolates was conducted using the VITEK^®^ 2 Compact system (bioMérieux, Marcy-l’Étoile, France). Pure colonies were suspended in sterile saline solution and adjusted to the manufacturer-recommended turbidity. The prepared bacterial suspensions were then inoculated into VITEK^®^ 2 antimicrobial susceptibility testing (AST) cards and processed following the manufacturer’s instructions.

The susceptibility profiles of the isolates were determined against a panel of 19 antimicrobial agents representing different antibiotic classes as shown in Table 1 with VITEK 2 system as described by Heine (2021) [14].

Minimum inhibitory concentrations (MICs) and susceptibility interpretations were determined automatically using the VITEK^®^ 2 software. Isolates were categorized as susceptible (S), intermediate (I), or resistant (R) based on the system’s interpretive criteria. We defined multidrug resistance as resistance to at least one antimicrobial agent in three or more antimicrobial classes. The Multiple Antibiotic Resistance (MAR) index, introduced by Krumperman in 1983 [15], was used to quantify the level of antimicrobial resistance in food isolates.

### 2.6. Molecular Identification of Escherichia coli Pathotypes

As part of routine testing at our laboratory, the molecular identification of *Escherichia coli* pathotypes was conducted using polymerase chain reaction (PCR) following the protocol outlined by Omar and Barnard (2014) [16].

#### 2.6.1. DNA Extraction

Cells from a 2 mL sample, pooled from 10 wells of the Quantri-tray, were collected by centrifugation (Eppendorf Centrifuge 5420; Sigma-Aldrich) at 13,000× *g* for two minutes. The supernatant was then discarded. DNA extraction from the bacterial cells was conducted using the silica thiocyanate method and an adapted spin method [16]. The adaptation involved adding 250 µL of 100% ethanol (Merck) to the lysis buffer to enhance DNA binding to Celite. In the spin method, Celite with bound DNA was transferred to a DNA-binding membrane (Corning CoStar Spin-X; Sigma-Aldrich). DNA was eluted using 100 µL of Qiagen elution buffer [17]. DNA quantification was performed using a Qubit Fluorometer following the manufacturer’s guidelines.

#### 2.6.2. PCR Identification of *E. coli* Pathotypes

Extracted DNA was subjected to multiplex PCR (mPCR) following the protocol described by Omar and Barnard (2010) [17]. Table 2 provides the specific primers used for *E. coli* pathotyping.

### 2.7. Molecular Identification of Enterobacter Species by 16S rRNA Gene Sequencing

*Enterobacter* species were identified and characterized at Inqaba Biotec (Pretoria, Gauteng, South Africa) using polymerase chain reaction (PCR), with primer details provided in Table 3. Targeted 16S amplicons were purified for sequencing using the Zymo Research ZR-96 DNA Sequencing Clean-up Kit™ (Catalogue No. D4050, Zymo Research, Irvine, CA, USA). DNA sequencing was conducted in both forward and reverse directions with the Nimagen BrilliantDye™ Terminator Cycle Sequencing Kit V3.1 (BRD3-100/1000, Thermo Fisher Scientific Inc., Waltham, MA, USA) on the ABI 3730xl Genetic Analyzer (Applied Biosystems, ThermoFisher Scientific; Waltham, MA, USA).

FinchTV (https://finchtv.software.informer.com/1.4/; version 1.4.0; Geospiza Inc., Seattle, WA, USA) (accessed on 14 June 2024) was utilized to view the raw chromatogram files (.abi). Subsequently, CLC Bio Main Workbench was employed to assemble forward and reverse reads into a consensus sequence for each sample. BLASTn analysis (default parameters) [24] was performed on the NCBI website (https://blast.ncbi.nlm.nih.gov/Blast.cgi) (accessed on 14 June 2024) to determine whether a database sequence matched the query sequence above the thresholds of 99% query coverage and 99% identity.

**Table 3:** Primers used for the identification of *Enterobacter* species.

	Primer	Sequence (5′-3′)	Reference
16S rDNA	*27 (F)*	AGA GTT TGA TCC TGG CTC AG	[25]
	*1492 (R)*	GGT TAC CTT GTT ACG ACT T	[25]

Footnote: 16S rDNA = 16S ribosomal DNA; F = forward primer; R = reverse primer; 5′-3′ = five-prime to three-prime direction.

### 2.8. Data Analysis

Statistical analyses were performed using Jamovi (Open Statistical Software, Version 2.6.3). Descriptive statistics were used to summarize the distributions of bacterial isolates, environmental parameters, and antimicrobial susceptibility profiles. The findings are presented in tables that show frequencies and percentages.

The Chi-square test of independence, Mann–Whitney U test, and Fisher’s exact test were applied to compare the distributions of bacterial species, *E. coli* pathotypes, and antimicrobial resistance across farms and sample types. Statistical significance was defined as *p* < 0.05.

## 3. Results

### 3.1. Physical Parameters of Irrigation Water

The irrigation water from all three farms exhibited acceptable electrical conductivity for irrigation but was slightly acidic and had TDS concentrations above the DWAF irrigation guideline (Table 4). Farm 3 (subsistence farm) consistently showed the highest average EC (373.67 µS/cm) and TDS (303.67 mg/L), and the lowest average pH, suggesting a comparatively higher mineral load and greater acidity than the other farms. The lowest EC and TDS were recorded in Farm 2. Concerning EC, values were below the irrigation minimum according to the DWAF, and the water did not meet drinking water quality standards, as per SANS and DWAF guidelines. The pH values were not adequate for irrigation and drinking use according to the DWAF, but they were acceptable according to the SANS guideline. These physicochemical characteristics may influence both the quality of irrigation water and the persistence of microbial contaminants on onions. Samples from river water were generally turbid by observation, compared to borehole water, and turbidity could not be measured.

Due to the limited number of sampling locations, inferential statistical comparison between the farms showed no significance.

### 3.2. Isolation and Identification of Total Coliform and E. coli in Onions and Irrigation Water Using Colilert Q-Tray

**Figure 2:**
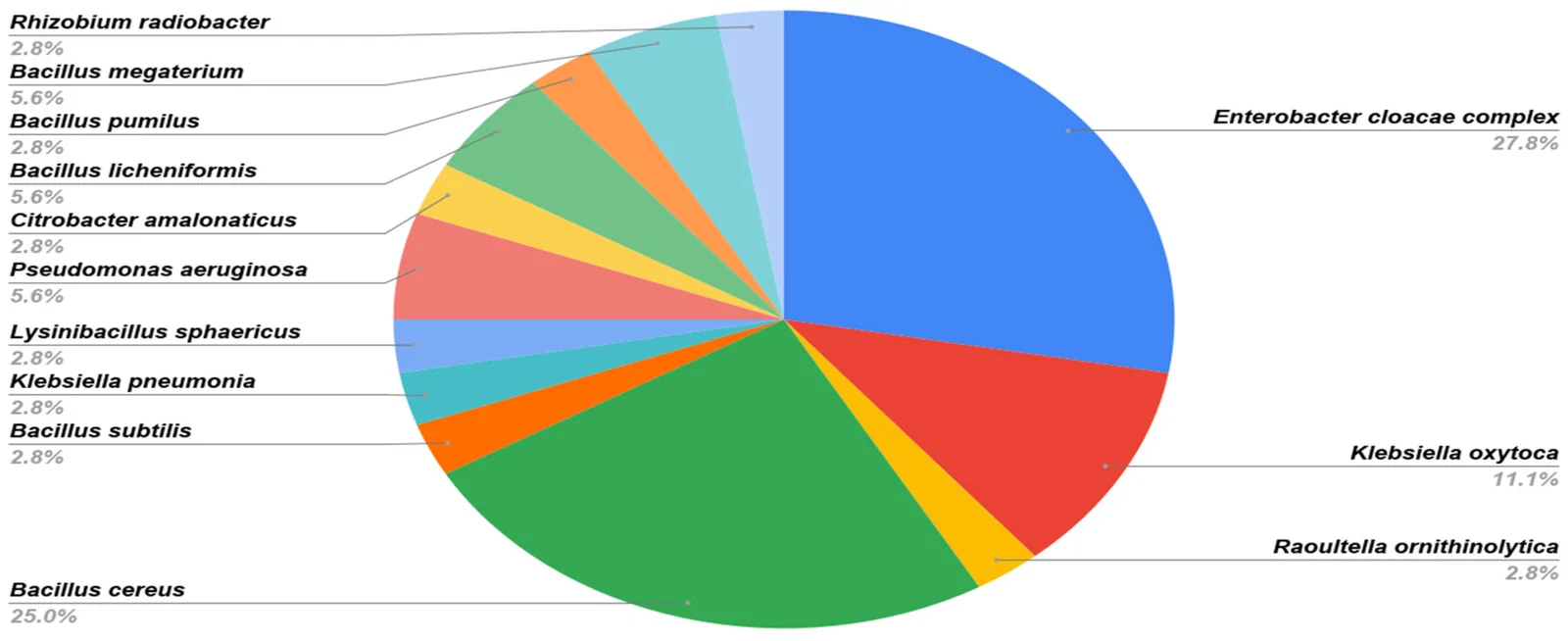
Frequency of various enteric and plant-associated pathogens detected by Vitek.

All samples were positive for total coliforms (TC), with counts exceeding 2419.6 MPN. One pooled onion sample from the commercial farm tested positive for *E. coli* (Table 5) but was confirmed as commensal *E. coli* by PCR. Irrigation water tested positive for TCs, with high counts like those found in onions. Several members of the Enterobacteriaceae family, forming part of the total coliforms, are shown in Figure 2. The PCR assay of commercial onion farm samples revealed that only commensal *E. coli* was present in onions from site A (Table 5). Further *E. coli* confirmation of onions from site B and the irrigation water, conducted by molecular biology techniques, detected no *E. coli*. Subsistent farms using river water for irrigation and the associated onions contained mixed pathotypes of *E. coli*, with the irrigation water in Farm 2 having the most diverse pathotypes.

*E. coli* MPN values in irrigation water and onion samples were not normally distributed; therefore, no significant difference was found among sample types (*p* = 0.414).

The association between pathogenic *E. coli* presence (Yes/No) and sample types was evaluated using Fisher’s Exact Test, as expected cell frequencies fell below the minimum threshold required for the Chi-Square test of Independence (χ^2^, *p* = 1.000).

### 3.3. Detection of Bacterial Isolates by Membrane Filtration

The presence of Enterobacter in the samples was detected using membrane filtration. We selected 36 representative colonies from onion (n = 13), irrigation water (n = 14), and soil (n = 9) samples based on distinct colony morphology on selective media. Colony morphology indicated the presence of a diverse population of enteric bacteria in onions, soil, and irrigation water. Onion samples yielded the highest number of presumptive isolates (31; 43.1%), followed by irrigation water (26; 36.1%) and soil (15; 20.8%).

After subculturing, most isolates (66.7%) were pink to red lactose-fermenting colonies (Table S1), predominantly associated with *Enterobacter*, *Klebsiella*, *Citrobacter*, and *Escherichia coli*. Metallic green sheen colonies characteristic of *E. coli* were detected exclusively in irrigation water (19.4%), while yellow/yellowish-pink colonies presumptively identified as *Proteus* spp. were found only in soil (8.3%). Brown mucoid colonies, suggestive of *Enterobacter* and *Klebsiella* spp., were common in onion and soil samples (69.4%). Other morphotypes included clear-white colonies suggestive of *Proteus* and *Providencia* spp. and purple colonies presumptively associated with *Enterobacter* spp. and *E. coli*.

### 3.4. Vitek Identification and Distribution of Bacterial Isolates

Gram staining confirmed 21 Gram-negative bacilli (GNB) and 15 Gram-positive bacilli (GPB), representing a diverse bacterial population. This revealed the presence of 13 bacterial species across irrigation sources, soil samples, and onions, as shown in Figure 2 and Table 6.

The most frequently confirmed pathogens included the Gram-negative *Enterobacter cloacae* complex (10/36, 27.8%), followed by *Bacillus cereus*, a Gram-positive bacterium (9/36, 25%), and *Klebsiella oxytoca* (4/36, 11.1%) (Figure 2). Other clinically significant pathogens, such as *K. pneumoniae* and *P. aeruginosa*, were detected in the soil of the commercial farm and one of the subsistence farms (Farm 3), respectively. *Rhizobium radiobacter*, a plant pathogen, was detected in the irrigation water for Farm 3 (Supplementary Table S1).

Bacterial distribution across farms (Table 6) was analysed using Fisher’s exact test (*p* = 0.080) after collapsing rare taxa (n < 3) into broader functional groups (Enterobacteriaceae, *Bacillus* spp., other Gram−, other Gram+) to satisfy the minimum expected cell count assumptions (χ^2^, *p* = 0.161).

**Figure 3:**
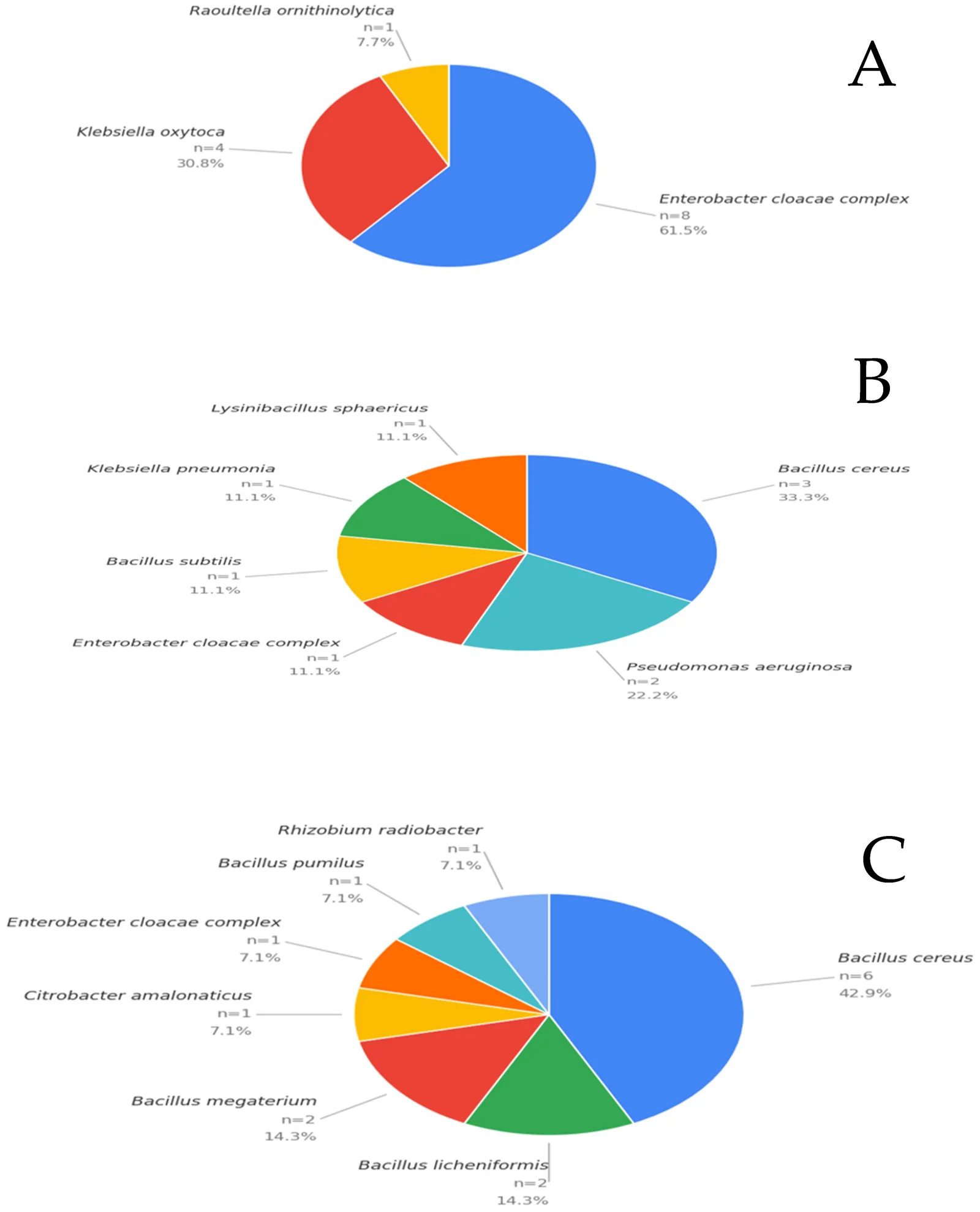
Frequency of various enteric and plant-associated pathogens in onions (**A**), soil (**B**), and all water samples (**C**). Onions contained three species, soil contained six species, and water samples had the most diversity, with seven species.

In Figure 3, onion samples were dominantly contaminated by *E. cloacae* complex (8/13; 61.5%) and *K. oxytoca* (4/13; 30.8%), while soil samples contained a greater diversity of species, including *B. cereus*, *B. subtilis*, *K. pneumoniae*, *Lysinibacillus sphaericus*, and *Pseudomonas aeruginosa*. Irrigation water samples were predominantly contaminated with *Bacillus* species, particularly *B. cereus* (6/14; 42.9%), as well as *Citrobacter amalonaticus*, *Enterobacter cloacae* complex, and *Rhizobium radiobacter*. The distribution of bacterial species varied among farms, with *E. cloacae* complex occurring in onions, soil, and irrigation water, indicating its widespread occurrence within the onion production environment.

### 3.5. Molecular Identification of Enterobacter Species by 16S rRNA Gene Sequencing

**Figure 4:**
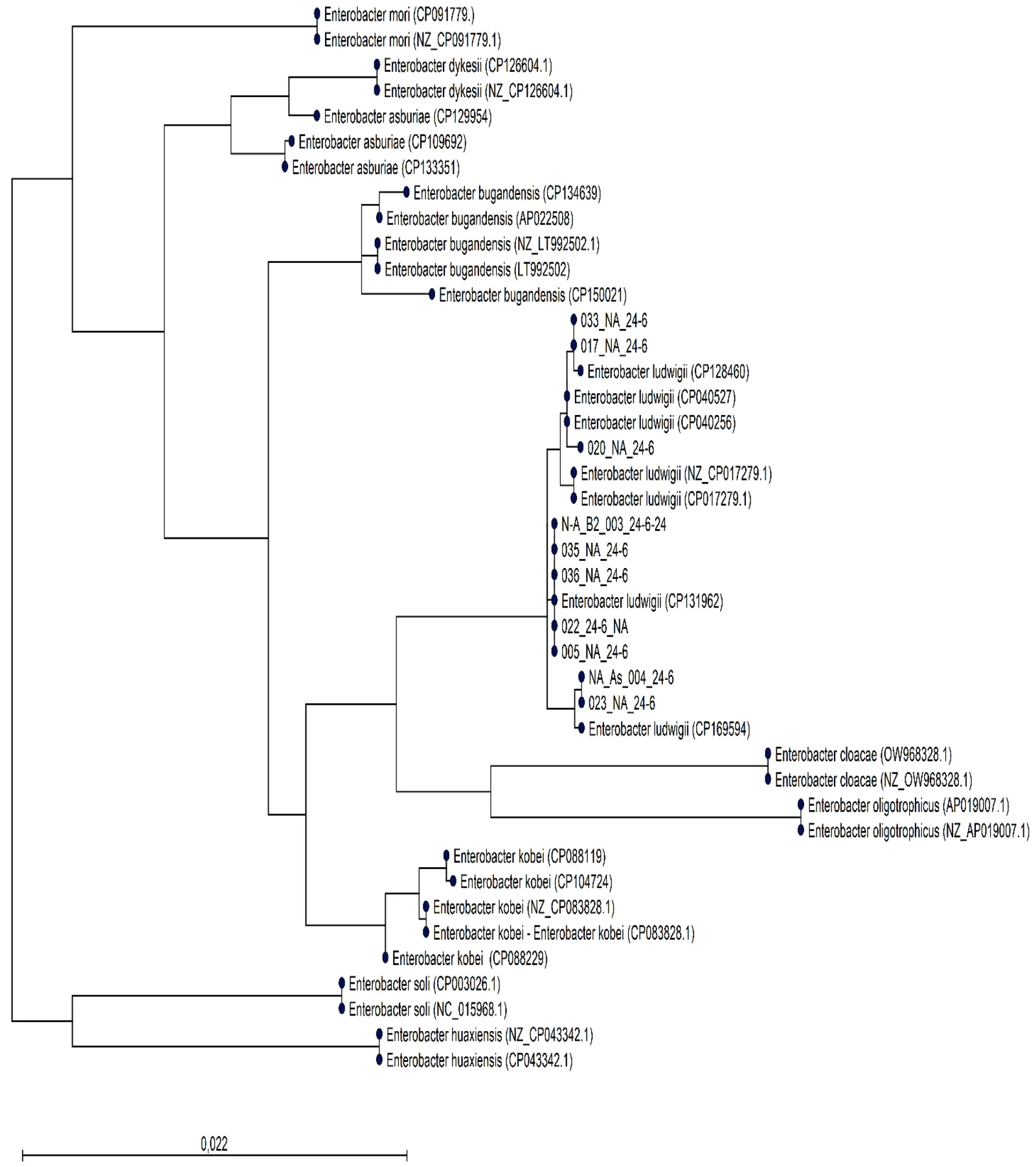
The phylogenetic tree based on the 16S target region (1.5 KB) of Enterobacter. We inferred the evolutionary history using a Neighbour-Joining tree of the Enterobacter gene (GenBank Accession numbers prefixed NZ and CP). Bootstrap values (>50%) are at branch nodes.

To identify which members of the *Enterobacter cloaceae* complex were present in the samples, 16S rRNA sequencing was performed, and Sanger sequence analysis using phylogenetic methods. Phylogenetic analysis of the 16S rRNA gene sequences showed that most isolates recovered in this study clustered within the *Enterobacter ludwigii* clade (Figure 4). The 10 *Enterobacter cloacae* complex isolates were predominantly recovered from onion samples (8/10), specifically A1, A2, A4, A5, B2, B3, B4, and OCE, with one isolate each recovered from a soil sample (S3 IWA) and a water sample (IWC 3W).All 10 Isolates grouped closely with previously characterized *E. ludwigii* reference strains, indicating a high degree of genetic similarity. The *E. ludwigii* cluster was clearly separated from other *Enterobacter* species included in the analysis, namely *E. bugandensis*, *E. asburiae*, *E. dykesii*, *E. mori*, *E. kobei*, *E. huaxiensis*, *E. cloacae*, *E. oligotrophicus*, and *E. soli*. The short branch lengths observed within the *E. ludwigii* cluster suggest limited genetic divergence among the isolates, whereas the distinct clustering of the remaining species confirms their phylogenetic separation. The 16S rRNA sequencing alone did not provide definitive species-level identification within closely related *Enterobacter* taxa.

### 3.6. Antibiotic Susceptibility of Identified Bacterial Species

**Figure 5:**
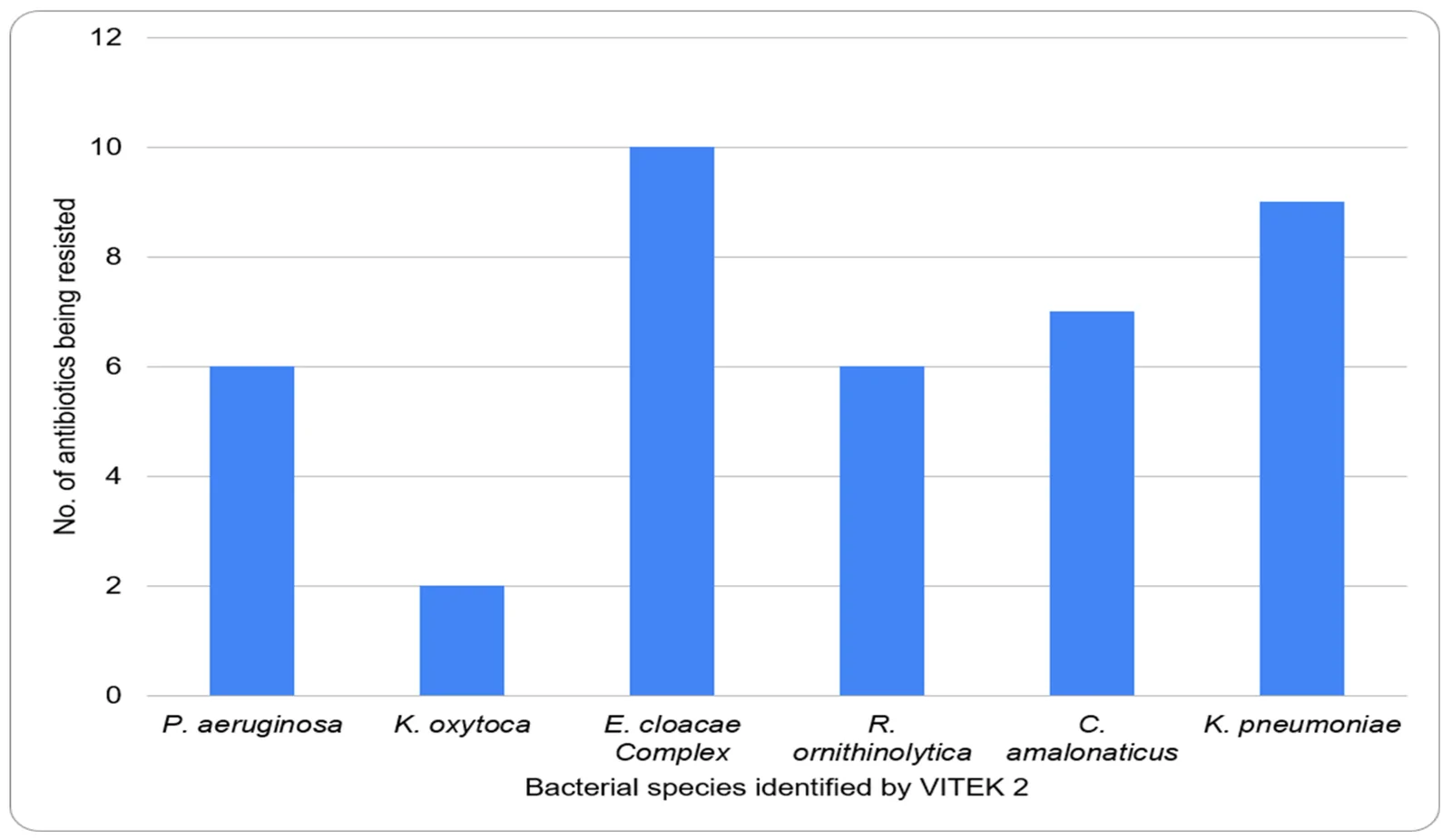
The comparison of the number of antibiotics being resisted by *P. aeruginosa*, *K. oxytoca*, *E. cloacae* Complex, *R. ornithinolytica*, *C. amalonaticus* and *K. pneumoniae*, based on the VITEK 2 19-antibiotic assay.

The antimicrobial susceptibility testing revealed variable resistance patterns among the six bacterial species tested against 19 antimicrobial agents (Table 7; Figure 5). The *Enterobacter cloacae* complex exhibited the highest level of resistance, showing resistance to 10 of the 19 antimicrobial agents tested (52.6%). This was followed by *Klebsiella pneumoniae* (9/19; 47.4%), *Citrobacter amalonaticus* (7/19; 36.8%), *Pseudomonas aeruginosa* and *Raoultella ornithinolytica*, each resistant to 6/19 agents (31.6%). *Klebsiella oxytoca* showed the lowest resistance, with resistance to only 2 of the 19 antibiotics tested (10.5%).

All six bacterial species showed resistance to colistin, while all isolates were susceptible to ciprofloxacin. Gentamicin also showed excellent activity, with all isolates remaining susceptible. Most isolates were susceptible to amikacin, although intermediate susceptibility was observed for *Enterobacter cloacae* complex and *Citrobacter amalonaticus*.

Among the β-lactam antibiotics, widespread resistance was observed to ampicillin, amoxicillin/clavulanic acid, cefuroxime, and cefuroxime axetil, particularly among Enterobacteriaceae. The *Enterobacter cloacae* complex was resistant to several cephalosporins, including cefotaxime and cefoxitin. In contrast, *Klebsiella pneumoniae* remained susceptible to cefotaxime, ceftazidime, and cefepime despite exhibiting resistance to several other β-lactams. *Pseudomonas aeruginosa* showed resistance to cefotaxime, ceftazidime, cefepime, tigecycline, and colistin but remained susceptible to aminoglycosides and ciprofloxacin.

Carbapenem susceptibility varied among the isolates. Meropenem remained effective against *Klebsiella oxytoca*, *Raoultella ornithinolytica*, and *Klebsiella pneumoniae*, whereas Pseudomonas aeruginosa, Enterobacter cloacae complex, and Citrobacter amalonaticus showed resistance or intermediate susceptibility. Similarly, most isolates exhibited intermediate susceptibility to imipenem and ertapenem, suggesting reduced carbapenem susceptibility in some species.

Finally, five of the six bacterial species exhibited multiple antibiotic resistance (MAR) indices greater than 0.20, indicating that these isolates likely originated from environments subjected to significant antimicrobial selective pressure (Table 8). Only *Klebsiella oxytoca* had a MAR index below 0.20 (0.11), suggesting comparatively lower antibiotic exposure. These results reinforce concerns about the spread of antimicrobial-resistant enteric bacteria via agricultural environments and irrigation water.

## 4. Discussion

This study identified *Enterobacter* species and other Enterobacteriaceae in onions, soil, and irrigation water in rural areas of the VDM. The water quality parameters affect irrigation systems, microbial activity, and water potability in various ways. Since the irrigation water sources for the backyard farms (Farm 2 and Farm 3) are also used for various activities beyond agriculture [9,11], domestic use guidelines were included to define irrigation water quality [26,27,28]. Total dissolved solids (TDS) all fall outside the guidelines for good irrigation quality but fall within acceptable standards for domestic use. Irrigation systems, microbes, and taste are all affected by the TDS composition. Increasing TDS levels pose a growing risk of clogging the dripper lines, thereby increasing the need for labour-intensive irrigation system maintenance. Guidelines suggest that controlling microbes within systems can help reduce TDS by preventing suspended solids from accumulating in biofilms. High levels of TDS and EC both contribute to the “saltiness” of the water, which negatively impacts it and, subsequently, the soil quality [26,28]. In drinking water, TDS predominantly affects aesthetics and taste up to 1000 mg/L, with a salty taste and appliances being affected. In this study, TDS values were well below this level, but due to the presence of other contaminants, they should be monitored, especially in rivers used for irrigation, and efforts to minimise pollution should be encouraged. Borehole water would be expected to be clear but can be brackish, rich in TDS and high in EC from natural lime in the soil, and thus it was fairly close in values to the river samples. The pH level at Farm 2 was just below the acceptable standard for irrigation water. Given that rivers serve as the irrigation source for backyard farms, the physicochemical properties observed in the Farm 2 samples diverge from those documented in previous studies of rivers in the Vhembe district [9,11]. According to the same guidelines, the pH should ideally be between 6.5 and 8.4 to prevent foliar damage, ensure the availability of macro- and micronutrients in the water, and prevent corrosion of irrigation equipment [29,30]. Further addition of parameters such as salinity and turbidity is encouraged for future studies to better understand the local drivers in microbial growth and survival.

Considering microbial pollution, the counts for both backyard farms were unacceptable for total coliforms (TCs) and *E. coli* in irrigation and drinking water [29]. The presence and concentrations of TCs and *E. coli* were similar to those reported in previous studies of river water in the Vhembe district. The most common pathotypes of *E. coli* in river water in VDM are atypical enteropathogenic *E. coli* (EPEC), enterotoxigenic *E. coli* (ETEC), and enteropathogenic *E. coli* (EPEC) [8,10]. Three recent studies have noted various contributors to river water quality in the area in recent years: Agricultural (animal grazing, irrigation, fishing, litter), human-related (laundry, bathing, swimming, washing of diapers), and infrastructure-related (domestic sewage) pollution seem to be widespread [8,11,29].

The detection of faecal pathogens, *E. coli* pathotypes and other Enterobacteriaceae, such as *Klebsiella*, is thus unsurprising given the documented burdens of river pollution, poor sanitation and diarrhoeagenic *E. coli* in the Vhembe District [10]. This underscores the urgent need for local communities and governance structures to claim stewardship over these precious water sources. Critically, the same rivers pose a documented persistent public health hazard and are underutilised water resources. Stable electricity supply in rural farm settings is crucial for equipment such as pumps drawing water from rivers on their farms. Micro-hydrokinetic river turbine (µ-HRT) technology, which generates electricity directly from river flow without dams or major infrastructure, has been identified as a viable low-tech solution for rural electrification in South Africa and across Africa [31]. Systems can be constructed from locally available or recycled materials, thereby reducing import costs and supporting local manufacturing [31]. Harnessing the Vhembe rivers for clean energy, while simultaneously monitoring and mitigating their microbiological contamination through ongoing food safety surveillance, could therefore contribute to achieving SDG 2 (zero hunger), SDG 3 (good health and wellbeing), SDG 6 (clean water and sanitation), and SDG 7 (affordable and clean energy) within a single integrated framework.

The borehole water from the commercial farm used for irrigation shows the presence of TCs but not *E. coli*. This differs from a recent widespread survey of borehole water in the VDM, which found *E. coli* in 87.5% and 72.5% of borehole samples in the wet and dry seasons, respectively [32]. A variety of factors have been identified as influencing groundwater quality in the Vhembe district, as indicated by local studies: the presence of animals, soil permeability, and runoff and seepage resulting from heavy rainfall [32,33,34]. This study is limited in that it did not sample over an extended period or sample from multiple boreholes on the farm.

Biochemical testing, such as VITEK 2, identified several bacterial species in water, soil, and onions. The dominant pathogenic species group, the *E. cloaceae* complex, is known to infect onions; it has been detected in other vegetables and irrigation water in South Africa, Africa, and other countries such as Iran. Additionally, other members of the Enterobacteriaceae family, such as *K. pneumoniae*, *K. oxytoca*, *E. coli*, and *Pantoea* spp., were present in this study and are also involved in various onion diseases [35,36,37,38]. This highlights the need for sensitive methods capable of distinguishing between pathogens. The sensitivity of PCR, combined with sequencing, was demonstrated in this study, which identified *E. ludwigii* as the specific member of the *E. cloacae* complex infecting onions at the pre-harvest stage. *Enterobacter* species are opportunistic pathogens responsible for a variety of diseases and exhibit a wide range of antibiotic resistance [39]. Broad-spectrum antibiotic resistance was present in this study, raising questions about its source.

The distinction of *E. ludwigii* was made in 2005 by Hoffman and colleagues [40], who observed that human isolates exhibited genetic and phenotypic characteristics distinct from those of other members of the *E. cloacae* complex. *E. ludwigii*, a member of the *Enterobacter cloacae* complex, has also been reported as a plant-associated bacterium and has been detected in onion tissues, where members of the *E. cloacae* complex have been linked to bulb decay and internal deterioration. These observations emphasized the necessity of precise identification of *Enterobacter* species associated with onion production and storage systems [41]. *Enterobacter ludwigii* can cause infections in neonates and may be resistant [42]. It was detected in carrots in the Limpopo province in 2023, revealing that *E. ludwigii* strains were closely related to strains from India and South Korea [43]. Another study designed 16S PCR primers to detect *macergens* in various rotting vegetables and discovered that two strains were closely related to *E. ludwigii* [43,44]. Although *E. ludwigii* is generally considered a plant pathogen, strain GMG_278 has been found to stimulate growth in the presence of mineral fertilizers through gibberellin production and oxidative stress [45]. Based on these observations, the presence of the *E. cloaceae* complex in irrigation water and soil suggests that irrigation water seeds the onions and the surrounding soil, with the onions providing a more favorable growth medium than the soil itself. Future investigations should conduct more rigorous studies, including increased replicates and broader sampling sites, together with phylogenetic analysis or 16S sequencing of all bacteria, to provide stronger evidence in future investigations. This is because other bacteria, such as the genus *Bacillus*, were also detected in high proportions in soil and irrigation water, but not in onions in this study (Figure 3). This raises questions about what makes onions targets for colonization and infection by Enterobacteriaceae such as *E. cloacae* and *Klebsiella*.

This study reports the presence of various *Bacillus* species (family Bacillaceae). These results are an oddity as media such as MacConkey typically inhibit Gram-positive bacteria. Growth of *Bacillus* species on these unconventional media has been reported: *B. cereus* has been grown on MacConkey agar during a case study of a pneumonia patient [46] and a patient from South Africa experiencing a rare case of *B. cereus*-linked septic arthritis [47]. A study by Apetroaie-Constantin and colleagues (2008) [48] indicates that *B. cereus* can grow well on certain media such as skim milk agar, raw milk agar, and MacConkey agar; the media affect the production of cereulide, an emetic, stable toxin. *Bacillus cereus* is a ubiquitous, spore-forming bacterium that can cause emetic or diarrhoeal illness in humans and is commonly linked to food poisoning through toxins. Spores enable *B. cereus* to survive in the environment, in animals and plants, and perhaps to grow on unconventional media. Other factors, however, such as biofilm formation and host plant properties, have a greater influence, which may explain its sparsity in the onions but abundance in soil and water [49]. A study by Motlhabatlou and colleagues [50] in South Africa indicated that similar *Bacillus* species, as well as *Klebsiella* species, have been found in urban water pipe biofilms. In experimental studies by Brillard and colleagues (2015) [51], *B. cereus* has been observed to survive in some groundwater samples, producing vegetative cells up to 50 days, albeit with low bacterial counts. They further note that spores are ubiquitous and are the more typical form present in agricultural soils and could assist in bacterial leaching through soils via percolated water after irrigation or rain. Ubiquity is sensible, especially in areas influenced by human pollution. Future studies could elaborate on the characteristics of *B. cereus* found within this setting and how it transmits through these environments [52]. Molecular detection techniques, such as PCR and sequencing, are sensitive in detecting antibiotic-resistant enteric pathogens in food and humans [53]. Kgoale and colleagues [37] found similar genera in leafy greens and irrigated soil using Illumina MiSeq high-throughput sequencing, such as *Klebiella*, *E. coli* and *Bacillus*, showing the potential for sequencing in agricultural settings in South Africa. Furthermore, methods that are equally sensitive but more rapid, such as gold-nanoparticle-based colourimetric tests, have been developed for the detection of an onion sour rot pathogen, *Burkholderia cepacia* [54], and could be evaluated for their use in the region with cost considerations in mind.

In this study, antibiotic susceptibility testing revealed that the *E. cloacae* complex harboured resistance to a wide variety of antibiotics. This genus is part of the ESKAPEE pathogens (*Enterococcus faecium*, *Staphylococcus aureus*, *Klebsiella pneumoniae*, *Acinetobacter baumannii*, *Pseudomonas aeruginosa*, *Enterobacter* species and *Escherichia coli*), recognised as critical, multidrug-resistant pathogens [55]. Antibiotic resistance in *E. ludwigii* is mostly recorded against extended-spectrum beta-lactams (ESBLs) and carbapenems, which is also noted in this study [56,57,58]. The presence of multidrug-resistant and ESBL-producing bacteria in food items could have major health implications if clinical infections caused by ESBL-producing enteric bacteria are not addressed swiftly. The region already contends with high levels of multidrug resistance and ESBL-producing enteric bacteria in its young population and suffers a high burden of diarrhoeal disease [14]. Interestingly, colistin resistance in enteric bacteria is widespread globally and has been identified in this study across all species tested in the antibiotic assay. Common species carrying colistin resistance genes, *mcr-*family, are *E. coli*, *K. pneumoniae*, *Salmonella enterica*, *P. aeruginosa* and *E. cloacae* [59], some of which were detected in this study. The genetic basis of resistance is poorly studied in the region, and future studies should determine which antibiotic resistance genes (ARGs) are circulating in water, crops, and soil. Phenotypic resistance to colistin in pathogenic bacteria such as *E. ludwigii* is alarming, as it is one of the last-line antibiotics for some severe bacterial infections. A recent study by Heine et al. (2024) identified a high frequency of ESBL genes in *E. coli* from young children under 5 in the VDM, highlighting the need for antibiotic surveillance in the region [60]. As a solution, antibiotic stewardship programs can serve as a starting point for reducing the frequency of antibiotic resistance, with potential applications in African countries, particularly through persuasive interventions such as education [61]. Farmers (both animal and vegetable), veterinarians, environmental officers and the human health sector could collaborate to address this growing issue. Establishing future surveillance and education initiatives to address such challenges, both in antibiotic management and river pollution, is therefore crucial.

A variety of factors influence the progression of diseases in onions, both during pre-harvest and post-harvest periods. During pre-harvest, factors such as the cultivar’s intrinsic resistance to the pathogen, the presence of animals, climate conditions (wet or dry seasons), disease vector control, nitrogen input from fertilisers, crop rotation, and seed and seedling health all play a role in the development of onion diseases. During post-harvest, curing temperature and moisture, and inoculation risk during handling or storage conditions play a role [62,63]. These factors could be examined in future studies on microbial detection and AMR of foodborne bacteria, thereby improving knowledge of onion longevity and food safety in the region, if challenges are identified and tackled strategically.

## 5. Conclusions

This study demonstrates the effectiveness of molecular, culturing and biochemical identification, revealing for the first time that *E. ludwigii* has been found on onions, in irrigation water, and in the soil of selected farms in the Vhembe District (Limpopo Province, South Africa). The bacteria were found exhibiting phenotypic resistance to various antibiotics, most notably ESBLs. The methods used revealed the presence of other *Enterobacteriaceae* and the known pathogen *Bacillus cereus* in the samples. An unconventional method, Colilert Quanti-Tray, was used to see if faecal bacteria can be detected on the onions themselves, showing detection of TCs and *E. coli*. This study confirms the persistent pollution of local water sources and calls for sustainable action by the local community and government. Further studies could expand on the number of farms being examined, using either groundwater or river water. Incorporating antibiotic resistance gene (ARG) discovery would strengthen these investigations, since the present study did not assess resistance at the genetic level. River water sources should be remediated to enable safer use in various human activities observed in the Vhembe District Municipality, including agriculture. Various pre- and post-harvest factors should be monitored in the region to control the spread of vegetable-associated pathogens.

## Figures and Tables

**Table 1:** Categories and concentration ranges of antimicrobials tested using the AST-N256 card.

Category	Antimicrobial	Range
Penicillins	Ampicillin	2–32 μg/mL
Penicillin and β-Lactam Inhibitors	Amoxicillin/Clavulanic acid	2/1–32/16 μg/mL
Anti-Pseudomonal Penicillins	Piperacillin/Tazobactam	4/4–128/4 μg/mL
Non-Extended Spectrum Cephalosporins	Cefuroxime	1–64 μg/mL
	Cefuroxime-Axetil	1–64 μg/mL
Cephalomycins	Cefoxitin	4–64 μg/mL
Extended Spectrum Cephalosporins	Cefotaxime	1–64 μg/mL
	Ceftazidime	1–64 μg/mL
	Cefepime	1–64 μg/mL
Carbapenems	Ertapenem	0.5–8 μg/mL
	Imipenem	0.25–16 μg/mL
	Meropenem	0.25–16 μg/mL
Aminoglycosides	Amikacin	2–64 μg/mL
	Gentamicin	1–16 μg/mL
	Tobramycin	1–16 μg/mL
Fluoroquinolones	Ciprofloxacin	0.25–4 μg/mL
Glycylcyclines	Tigecycline	0.5–8 μg/mL
Polymixins	Colistin	0.5–16 μg/mL
Folate pathway inhibitors	Trimethoprim/Sulfamethoxazole	20–320 μg/mL

**Table 2:** Primers used for the identification and pathotyping of *E. coli*.

Pathotype	Primer	Sequence (5′-3′)	Reference
*E. coli*	*mdh (F)*	GGT ATG GAT CGT TCC GAC CT	[18]
	*mdh (R)*	GGC AGA ATG GTA ACA CCA GAG T	
EIEC	*ial (F)*	GGT ATG ATG ATG ATG AGT CCA	[19]
	*ial (R)*	GGA GGC CAA CAA TTA TTT CC	
EHEC/Atypical EPEC	*eaeA (F)*	CTG AAC GGC GAT TAC GCG AA	[20]
	*eaeA (R)*	CCA GAC GAT ACG ATC CAG	
Typical EPEC	*bfpA (F)*	AAT GGT GCT TGC GCT TGC TGC	[21]
	*bfpM (R)*	TAT TAA CAC CGT AGC CTT TCG CTG AAG TAC CT	[15]
EAEC	*eagg (F)*	AGA CTC TGG CGA AAG ACT GTA TC	[20]
	*eagg (R)*	ATG GCT GTC TGT AAT AGA TGA GAA C	
EHEC	*stx1 (F)*	ACA CTG GAT GAT CTC AGT GG	[21]
	*stx1 (R)*	CTG AAT CCC CCT CCA TTA TG	
	*stx2 (F)*	CCA TGA CAA CGG ACA GCA GTT	[21]
	*stx2 (R)*	CCT GTC AAC TGA GCA CTT TG	
ETEC	*lt (F)*	GGC GAC AGA TTA TAC CGT GC	[21]
	*lt (R)*	CGG TCT CTA TAT TCC CTG TT	
	*st (F)*	TTT CCC CTC TTT TAG TCA GTC AAC TG	[21]
	*st (R)*	GGC AGG ATT ACA ACA AAG TTC ACA	
*E. coli* toxin	*astA (F)*	GCC ATC AAC ACA GTA TAT CC	[22]
	*astA (R)*	GAG TGA CGG CTT TGT AGT C	
External control	*gapdh (F)*	GAG TCA ACG GAT TTG GTC GT	[23]
	*gapdh (R)*	TTG ATT TTG GAG GGA TCT CG	

Footnote: *E. coli* = *Escherichia coli*; EIEC = Enteroinvasive *Escherichia coli*; EHEC = Enterohemorrhagic *Escherichia coli*; EPEC = Enteropathogenic *Escherichia coli*; EAEC = Enteroaggregative *Escherichia coli*; ETEC = Enterotoxigenic *Escherichia coli*; F = forward primer; R = reverse primer; *mdh* = malate dehydrogenase gene; *ial* = invasion-associated locus; *eaeA* = *E. coli* attaching and effacing gene A; *bfpA* = bundle-forming pilus gene A; *bfpM* = bundle-forming pilus gene M; *eagg* = enteroaggregative *E. coli* heat-stable enterotoxin-associated gene; *stx1* = Shiga toxin 1 gene; *stx2* = Shiga toxin 2 gene; *lt* = heat-labile enterotoxin gene; *st* = heat-stable enterotoxin gene; *astA* = enteroaggregative *E. coli* heat-stable enterotoxin 1 gene; *gapdh* = glyceraldehyde-3-phosphate dehydrogenase gene; 5′-3′ = five-prime to three-prime direction.

**Table 4:** The average physical parameter values of irrigation water used for onions in the Vhembe region.

Sampling Sites	Temperature (°C)	Electrical Conductivity (EC) (µS/cm)	pH	TDS (mg/L)
Farm 1	21.73	245	6.24	141.53
Farm 2	23.9	214.33	6.29	136.53
Farm 3	20	373.67	5.63	303.67
DWAF Standard (irrigation) [26]	N/A	<540	[6.5–8.5]	<50
DWAF Standard (domestic use) [27]	N/A	0–70	[6.5–8.5]	<0–450
SANS Guideline (Drinking) [28]	N/A	<170	[5–9.7]	<1200

Footnote: EC = electrical conductivity; TDS = total dissolved solids; DWAF = Department of Water Affairs and Forestry; SANS = South African National Standard; N/A = not applicable; µS/cm = microsiemens per centimetre; mg/L = milligrams per litre; °C = degrees Celsius.

**Table 5:** Quantity and pathotype characteristics of *E. coli* in irrigation water and onion quality using Colilert Quanti-Tray with confirmation from a multiplex PCR assay.

	Total Coliforms (MPN/100 mL)	*E. coli* (MPN/100 mL)	Interpretation	PCR Confirmation and Pathotypes
**Sample ID Farm 1**				
Onions site A	2419.6	1.00	Low-level *E. coli* contamination; PCR identified commensal *E. coli*.	Commensal
Onions site B	2419.6	0	Total coliforms present but no detectable *E. coli*.	No *E. coli*
Irrigation water	2419.6	0	Total coliforms present but no detectable *E. coli*.	No *E. coli*
**Sample ID Farm 2**				
Onions 1	2419.6	100.5	High-level *E. coli* contamination; PCR identified pathogenic *E. coli*.	EPEC/ETEC
Onions 2	2419.6	100.5	High-level *E. coli* contamination; PCR identified pathogenic *E. coli*.	EPEC/ETEC/EAEC
Irrigation water	2419.6	200.5	High-level *E. coli* contamination; PCR identified pathogenic *E. coli*.	EPEC/ETEC/EIEC/EAEC
**Sample ID Farm 3**				
Onions 1	2419.6	100.5	High-level *E. coli* contamination; PCR identified pathogenic *E. coli*.	EPEC
Onions 2	2419.6	100.5	High-level *E. coli* contamination; PCR identified pathogenic *E. coli*.	EPEC/ETEC/EAEC
Irrigation water	2419.6	200.5	High-level *E. coli* contamination; PCR identified pathogenic *E. coli*.	EPEC/ETEC

Footnote: MPN = most probable number; PCR = polymerase chain reaction; *E. coli* = *Escherichia coli*; EPEC = enteropathogenic *Escherichia coli*; ETEC = enterotoxigenic *Escherichia coli*; EIEC = enteroinvasive *Escherichia coli*; EAEC = enteroaggregative *Escherichia coli*; mL = millilitre.

**Table 6:** Vitek identification and distribution of bacterial isolates.

	Farm 1			Farm 2			Farm 3			Total
	Onions	Soil	Water	Onions	Soil	Water	Onions	Soil	Water	
*Enterobacter cloacae* complex	4	1	1	3			1			10
*Klebsiella oxytoca*	1			1			2			4
*Raoultella ornithinolytica*							1			1
*Bacillus cereus*		3	2			2			2	9
*Bacillus subtilis*		1								1
*Klebsiella pneumoniae*					1					1
*Lysinibacillus sphaericus*					1					1
*Pseudomonas aeruginosa*								2		2
*Citrobacter amalonaticus*			1							1
*Bacillus licheniformis*						1			1	2
*Bacillus pumilus*						1				1
*Bacillus megaterium*						1			1	2
*Rhizobium radiobacter*									1	1
Total	5	5	4	4	2	5	4	2	5	36

**Table 7:** Antimicrobial susceptibility profiles of Gram-negative bacterial isolates as determined by the VITEK^®^ 2 Compact system.

Antimicrobial Agents	Antibiotic Class	*Pseudomonas aeruginosa* (MIC)	*Klebsiella oxytoca* (MIC)	*Enterobacter cloacae* Complex (MIC)	*Raoultella ornithinolytica* (MIC)	*Citrobacter amalonaticus* (MIC)	*Klebsiella pneumoniae* (MIC)
**Ampicillin**	β-Lactam, Penicillin	**-**	**R >= 32**	**-**	**R >= 32**	**-**	**R >= 32**
**Imipenem**	β-Lactam, Carbapenem	**-**	**I 2**	**I 2**	**I 2**	**I 2**	**I 2**
**Amoxicillin/Clavulanic Acid**	β-Lactam, Penicillin + β-Lactamase inhibitor	**-**	**I 16**	**R >= 32**	**R >= 32**	**R >= 32**	**R16**
**Piperacillin/Tazobactam**	β-Lactam, Penicillin + β-Lactamase inhibitor	**-**	**S <= 4**	**R >= 128**	**S 8**	**R64**	**R <= 4**
**Cefuroxime**	β-Lactam, Second-generation cephalosporin	**-**	**I 16**	**R >= 64**	**R >= 64**	**R >= 64**	**R >= 64**
**Cefuroxime Axetil**	β-Lactam, Second-generation cephalosporin	**-**	**I 16**	**R >= 64**	**R >= 64**	**R >= 64**	**R >= 64**
**Cefoxitin**	β-Lactam, Second-generation cephalosporin	**-**	**S <= 4**	**R >= 64**	**R >= 64**	**S <= 4**	**S <= 4**
**Cefotaxime**	β-Lactam, Third-generation cephalosporin	**R >= 64**	**-**	**R8**	**I 2**	**R >= 64**	**R <= 1**
**Ceftazidime**	β-Lactam, Third-generation cephalosporin	**R >= 64**	**S 2**	**I 16**	**I 8**	**R16**	**R <= 1**
**Cefepime**	β-Lactam, Fourth-generation cephalosporin	**R >= 64**	**S <= 1**	**I 4**	**I 4**	**I 8**	**R <= 1**
**Ertapenem**	Beta-lactam, Carbapenem class	**-**	**S <= 0.5**	**I 1**	**I 1**	**I 1**	**I 1**
**Meropenem**	Beta-lactam, Carbapenem class	**I 4**	**S 1**	**R < 1**	**S 1**	**R > 4**	**S 1**
**Amikacin**	Aminoglycoside	**S 4**	**S 8**	**I 8**	**S 4**	**I 32**	**S 8**
**Gentamicin**	Aminoglycoside	**S <= 1**	**S 2**	**S 2**	**S <= 1**	**S 4**	**S <= 1**
**Tobramycin**	Aminoglycoside	**S <= 1**	**S 2**	**R <= 2**	**S <= 1**	**S 4**	**S <= 1**
**Ciprofloxacin**	Fluoroquinolone	**S <= 0.25**	**S <= 0.25**	**S <= 0.25**	**S <= 0.25**	**S <= 0.25**	**S <= 0.25**
**Tigecycline**	Tetracycline	**R >= 8**	**S <= 0.5**	**S <= 0.5**	**S <= 0.5**	**S <= 0.5**	**S <= 0.5**
**Colistin**	Polymyxin	**R >= 8**	**R >= 64**	**R >= 64**	**R >= 64**	**R >= 64**	**R >= 64**
**Trimethoprim/Sulfamethoxazole**	Sulfonamide + dihydrofolate reductase	**-**	**S <= 20**	**S <= 20**	**S <= 20**	**S <= 20**	**S <= 20**
Total resistances	-	6/19	2/19	10/19	6/19	7/19	9/19

S = susceptible; I = intermediate/susceptible, increased exposure, as applicable according to the CLSI breakpoint; R = resistant; MIC = minimum inhibitory concentration (µg/mL). MIC values were interpreted using the CLSI M100 breakpoint criteria applicable to each bacterial species and antimicrobial agent. Red highlights indicate antimicrobial resistance.

**Table 8:** Antimicrobial susceptibility profile, resistance percentages, and Multiple Antibiotic Resistance (MAR) indices of bacterial isolates recovered from onions, soil, and irrigation water in the VDM, South Africa.

Bacterial Species	No of Antibiotics Tested	No of Resistant	Resistance %	MAR Index	Resistance Interpretation
*Pseudomonas aeruginosa*	19	6	31.6	0.32	High resistance
*Klebsiella oxytoca*	19	2	10.5	0.11	Low resistance
*Enterobacter cloacae* Complex	19	10	52.6	0.53	Very high resistance
*Raoultella ornithinolytica*	19	6	31.6	0.32	High resistance
*Citrobacter amalonaticus*	19	7	36.8	0.37	High resistance
*Klebsiella pneumoniae*	19	9	47.4	0.47	Very high resistance

MAR Index = Number of antibiotics to which the isolate was resistant ÷ Total number of antibiotics tested (19).

## Data Availability

The original contributions presented in this study are included in the article/Supplementary Materials. Further inquiries can be directed to the corresponding author.

## Supplementary Materials

The following supporting information can be downloaded at: https://www.mdpi.com/article/10.3390/foods15173046/s1, Table S1: Presumptive identified bacterial isolated by sample types; Table S2: Vitek-2 identification of Gram-stained isolates.
