# Supplementary material for: First Report of Enterobacter ludwigii and Other Potentially Pathogenic Enteric Bacteria in Onions, Soil, and Irrigation Water from the Vhembe Region, South Africa
Source: Foods. 2026 Aug 28;15(17):3046. doi: 10.3390/foods15173046 (PMC13564731; doi:10.3390/foods15173046)
Supplement: Supplementary file 1 [file foods-15-03046-s001.zip › foods-4503316-supplementary.pdf]

<u>Table S1.</u> Presumptive identified bacterial isolated by sample types

Source	Colour and Morphology	Presumptive bacterial identification
Onion	Pink, mucoid, and moist	*Citrobacter, Klebsiella spp, Enterobacter spp*
Onion	Pink, mucoid, and moist	*Citrobacter, Klebsiella spp, Enterobacter spp*
Onion	Pink with a dot and slight rough	*Enterobacter spp*
Onion	Pink mucoid, and moist	*Enterobacter, Klebsiella spp., Citrobacter spp*
Onion	Pink mucoid, and moist	*Enterobacter, Klebsiella spp., Citrobacter spp*
Onion	Pink and mucoid	*Citrobacter, Klebsiella spp*
Soil	Yellow	*Proteus spp*
Water	Pink, smooth,	*Enterobacter spp, Klebsiella spp*
Water	Pink, red with a dot, smooth-moist	*Citrobacter, spp, E. coli, Enterobacter spp*
Water	Pink	*E. coli*
Water	Pink	*E. coli*
Water	clear with a dot	*Hafnia*
Water	Pink, mucoid, and moist	*Klebsiella spp, Enterobacter spp, Citrobacter spp*
Water	Metallic green sheen	*E. coli*
Water	Pink with a dot and slight rough	*Enterobacter spp, klebsiella, Citrobacter spp*
Soil	Pink, smooth and mucoid	*Klebsiella spp, Citrobacter spp*
Onion	Brown with mucous texture	*Enterobacter spp, klebsiella*
Water	Metallic green sheen	*E. coli*
Soil	Pink with a dot	*E. coli*
Onion	Pink mucoid, and moist	*Enterobacter spp Klebsiella spp., Citrobacter spp*
Soil	Pink, red with a dot, smooth-moist	*Citrobacter, spp, E. coli, Enterobacter spp*
Onion	Brown with mucous texture	*Enterobacter spp, klebsiella*
Onion	Dark pink	*Klebsiella spp, Enterobacter spp, E. coli*
Onion	Pink, smooth and mucoid	*Enterobacter spp, klebsiella*
Water	Pink with a dot	*E. coli*
Soil	Yellow	*Proteus spp*
Soil	Pink and mucoid	*Citrobacter, Klebsiella spp*
Water	Pink with a dot and slight mucoid	*Klebsiella spp, Enterobacter spp*

Water	Purple	*Enterobacter spp, E. coli*
Soil	Yellowish pink	*Proteus spp*
Soil	Clear-White	*Proteus spp, Providencia spp*
Water	Pink, large, mucoid with red pigment	*E. coli, Citrobacter spp, klebsiella spp*
Water	Pink with white dot, smooth- moist	*Klebsiella spp, Enterobacter spp*
Onion	Brown with mucous texture	*Enterobacter spp, klebsiella*
Onion	Brown with mucous texture	*Enterobacter spp, klebsiella*
Soil	Brown with mucous texture	*Enterobacter spp, klebsiella*

Footnote: *spp. = species (plural); E. coli = Escherichia coli. All bacterial genus and species names are italicized according to standard bacterial nomenclature.* ***Note:*** *The organisms listed under "Presumptive bacterial identification" represent presumptive identifications based on colony colour and morphology and should not be interpreted as confirmed species-level identifications.*

Table S2. Vitek-2 identification of Gram-stained isolates

Code	Univen ID	Gram rxn	Detected Pathogens
Onions sample prevalence			
23	Onion A2	GNB	*Enterobacter Cloacae Complex*
35	Onion A1	GNB	*Enterobacter Cloacae Complex*
34	Onion A3	GNB	*Klebsiella oxytoca*
5	Onion A4	GNB	*Enterobacter Cloacae Complex*
4	Onion A5	GNB	*Enterobacter Cloacae Complex*
3	Onion B2	GNB	*Enterobacter Cloacae Complex*
17	Onion B3	GNB	*Enterobacter Cloacae Complex*
22	Onion B4	GNB	*Enterobacter Cloacae Complex*
24	Onion B5	GNB	*Klebsiella oxytoca*
1	OBM	GNB	*Klebsiella oxytoca*
2	OBE	GNB	*Klebsiella oxytoca*
6	OCM	GNB	*Raoultella Ornithinolytica*
20	OCE	GNB	*Enterobacter Cloacae Complex*
Soil sample prevalence			
16	S2 IWA	GPB	*Bacillus Cereus*
26	IWA S3	GPB	*Bacillus Cereus*
27	IWA S1	GPB	*Bacillus Cereus*
36	S3 IWA	GNB	*Enterobacter Cloacae Complex*
31	SI IWA	GPB	*Bacillus subtilis*
19	SBE	GNB	*Klebsiella pneumonia*
30	SBM	GPB	*Lysinibacillus sphaericus*
21	SCM	GNB	*Pseudomonas aeruginosa*
7	SCE	GNB	*Pseudomonas aeruginosa*
Water sample prevalence			
11	BHW	GPB	*Bacillus Cereus*
18	IWA W1	GNB	*Citrobacter amalonaticus*
33	IWC 3W	GNB	*Enterobacter Cloacae Complex*
28	IWB W2	GPB	*Bacillus Cereus*
12	FB3M water	GPB	*Bacillus Cereus*
13	FBME water	GPB	*Bacillus licheniformis*
29	FB2M water	GPB	*Bacillus Cereus*
25	FB2E water	GPB	*Bacillus Pumilus*
32	FB1E water	GNB	*Bacillus megaterium*
14	FC2M water	GPB	*Bacillus Cereus*
15	FCMM water	GPB	*Bacillus Cereus*
8	FCME water	GNB	*Rhizobium radiobacter*
9	FC3E water	GPB	*Bacillus licheniformis*
10	FC1E water	GPB	*Bacillus megaterium*
